# Persistent Hematuria With Late Diagnosis of Cardiac Origin: A Case Report

**DOI:** 10.7759/cureus.88069

**Published:** 2025-07-16

**Authors:** Nicholas Hamilton, Shipra Hingorany, Michael Mazar

**Affiliations:** 1 Medicine, University of California Los Angeles (UCLA) David Geffen School of Medicine, Los Angeles, USA; 2 Cardiology, University of California Los Angeles (UCLA) David Geffen School of Medicine, Los Angeles, USA

**Keywords:** hematuria, mechanical mitral valve complications, paravalvular leak closure, paravalvular leak (pvl), prosthetic valve dysfunction

## Abstract

Dark-colored urine is often referred to as hematuria; however, this is an assumption that can often be misleading. In the evaluation of dark-colored urine, distinguishing between true hematuria, hemoglobinuria, and myoglobinuria is essential, as each indicates different underlying pathologies and requires targeted diagnostic and management strategies. Visualization of dark-colored urine often leads to an isolated urologic evaluation, and assessment for more infrequent causes of this phenomenon may be obscured. Clinicians tend to anchor on common urologic causes of dark-colored urine or rely on dipstick analysis alone, thus failing to perform a more systemic evaluation. We present a case of persistent hematuria with cardiac origins that remained undiagnosed for many months. A detailed history and attention to hematologic indices should be pursued when urologic causes of hematuria prove to be unrevealing.

## Introduction

The differential diagnosis for dark-colored urine is broad and often challenging, as it encompasses a range of benign to life-threatening conditions. Common causes, such as hematuria, hemoglobinuria, and myoglobinuria, must be differentiated from less frequent causes, including porphyria, alkaptonuria, and medication-induced hematuria. Hemolysis results in the release of free hemoglobin into the circulation, which, when filtered by the kidneys, leads to hemoglobinuria and the clinical finding of dark-colored urine [1]. While numerous conditions can cause hemolysis, prosthetic valve dysfunction represents a rare but potentially catastrophic etiology [2].

Prosthetic valve dysfunction can manifest as obstruction or regurgitation. Prosthetic valve regurgitation may occur through the valve leaflets, which can be intravalvular or transvalvular, and paravalvular (also referred to as paravalvular leak (PVL)) [3]. Dark-colored urine can be a result of sheer stress on red blood cells (RBCs), resulting in hemolysis and hemoglobinuria. This is a rare presentation of prosthetic valve dysfunction that may remain undiagnosed if not assessed properly. Identification of prosthetic valve dysfunction is paramount as it is associated with poor outcomes. In this report, we describe a case of PVL presenting as dark urine.

## Case presentation

A 71-year-old female patient with a past medical history of severe rheumatic mitral stenosis status post St. Jude mechanical mitral valve replacement (MVR), paroxysmal valvular atrial fibrillation status post maze procedure, intracranial hemorrhage, and hypertension, presented to the emergency department (ED) for expedited evaluation of recurrent hematuria with resulting symptomatic anemia.

The patient first reported dark-colored urine to her primary care physician (PCP) at an outside institution, three months prior to the presentation at the ED. Urinalysis results from PCP, if obtained, were not available for review. She was then referred to urology at the same institution. The urologic evaluation included CT abdomen, cystoscopy, and fluorescence in situ hybridization (FISH) bladder screening. All the studies were unremarkable. The etiology of her hematuria remained unknown. Outpatient labs demonstrated severe anemia with hemoglobin (Hgb) 7 g/dL, and she underwent transfusion of two units of packed red blood cells (pRBC). Warfarin was held for 48 hours, but hematuria persisted, so it was resumed. She subsequently established care with urology at our institution. This evaluation included repeat cystoscopy, which was again unrevealing. During this time, she experienced worsening fatigue and dyspnea on exertion. Review of symptoms was negative for hematemesis, hematochezia, melena, or vaginal bleeding. The patient experienced another decline in Hgb to 6.8 g/dL and was referred to our ED.

On presentation at the ED, the patient's blood pressure was 158/64, heart rate was 74 beats/minute (bpm), and oxygen saturation was 94% on room air. On exam, she appeared mildly jaundiced. Auscultation demonstrated a mechanical S1 and 3/6 holosystolic murmur at the cardiac apex.

Blood tests showed normocytic anemia with elevated total and unconjugated bilirubin levels. Iron studies were completed post transfusion and thus uninterpretable. Additional anemia labs were significant for hemolysis (Table 1). Coomb’s test was negative. Peripheral smear demonstrated schistocytes. Urinalysis demonstrated 2+ blood and 13 RBC. Urine culture was negative.

**Table 1:** Blood laboratory tests MCV: mean corpuscular volume; RDW: red cell distribution width; AST: aspartate aminotransferase; ALT: alanine aminotransferase; PT: prothrombin time

Parameters	Reference ranges	Patient values
Hemoglobin	11.6-15.2 g/dL	6.8 g/dL
Hematocrit	34.9-45.2%	21.9%
MCV	79.3-98.6 fL	94.4 fL
RDW	11.1-15.5%	22.4%
AST	13-47 U/L	161 U/L
ALT	8-64 U/L	48 U/L
Total bilirubin	0.1-1.2 mg/dL	1.6 mg/dL
Conjugated bilirubin	<0.3 mg/dL	0.2 mg/dL
Lactate dehydrogenase	125-256 U/L	1964 U/L
PT	11.5-14.4 seconds	23.7 seconds
Reticulocyte count	No reference range	8%
Immature reticulocyte fraction	2.6-15.8%	27.8%
Haptoglobin	21-210 mg/dL	<8 mg/dL

**Figure 1:**
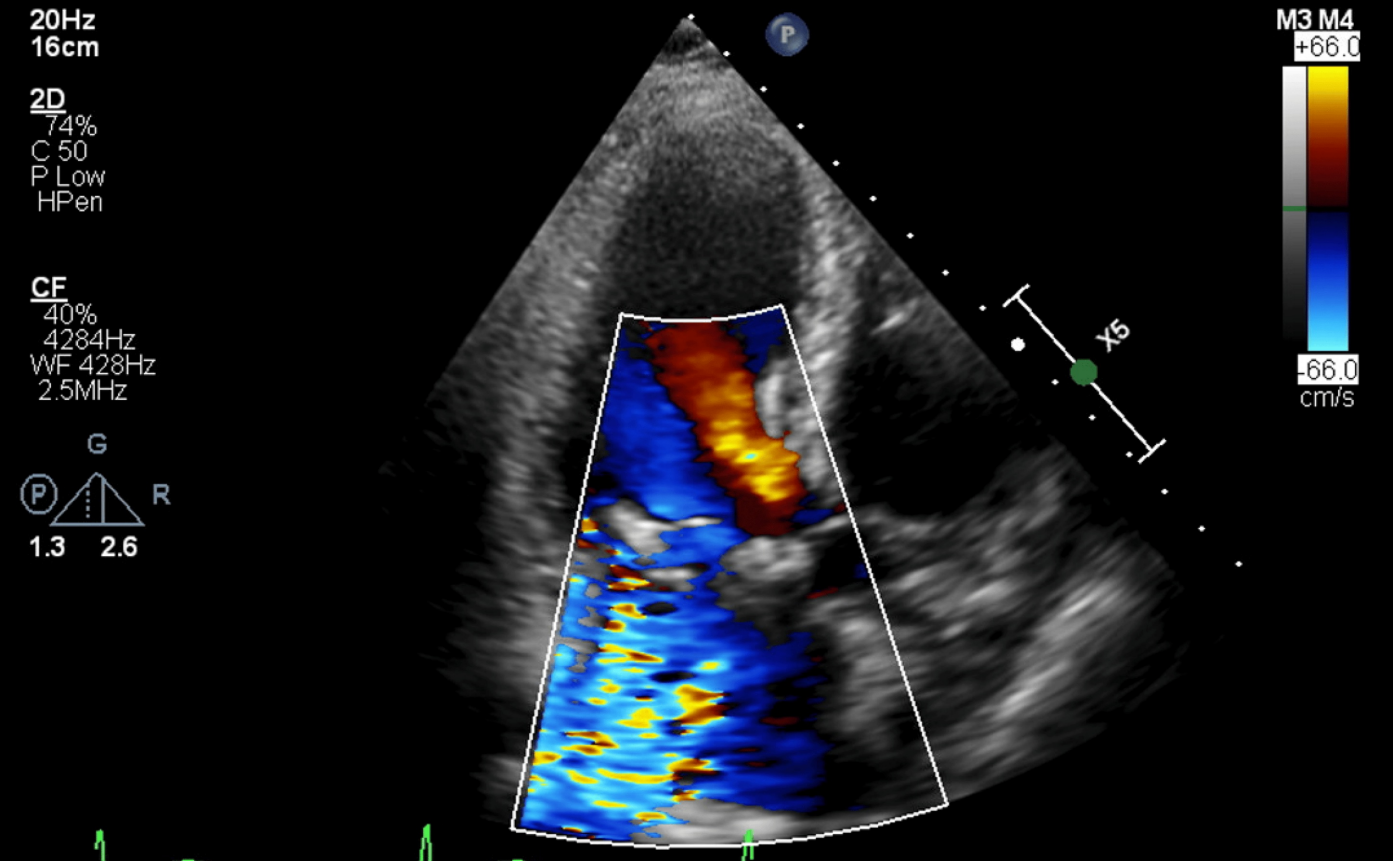
Transthoracic echocardiogram apical three-chamber view demonstrating paravalvular regurgitation.

Transthoracic echocardiography (TTE) was advised on admission, given the presence of a mechanical mitral valve and a holosystolic murmur noted on exam. TTE demonstrated a mechanical valve prosthesis present in the mitral valve position. The mechanical valve prosthesis did not appear to be rocking or demonstrate instability. There was moderate PVL noted (Figure 1) with a transmitral mean gradient of 6 mmHg at a heart rate of 76 bpm.

**Figure 2:**
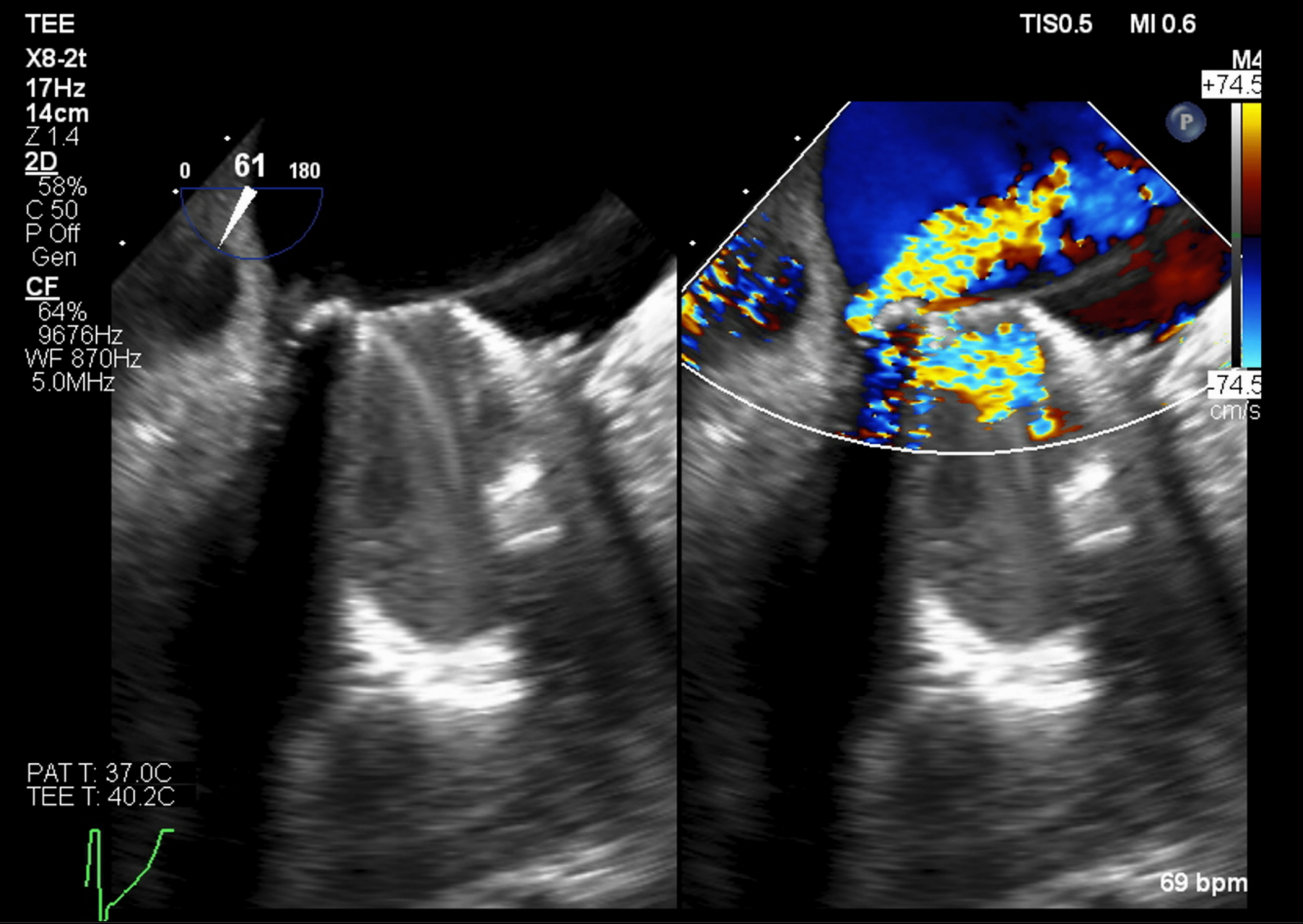
Transesophageal echocardiogram mid-esophageal mitral commissural view with and without color Doppler, demonstrating paravalvular regurgitation originating from the posterior aspect of the mitral valve prosthesis.

**Figure 3:**
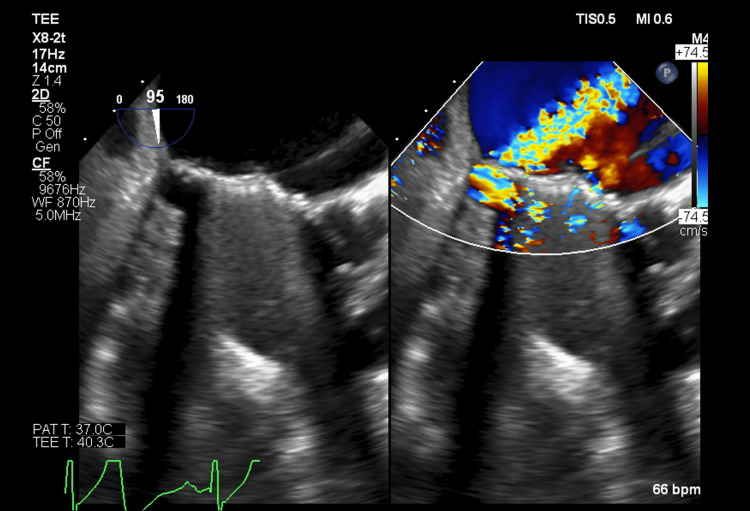
Transesophageal echocardiogram mid-esophageal two-chamber view with and without color Doppler, demonstrating paravalvular regurgitation originating from the posterior aspect of the mitral valve prosthesis.

**Figure 4:**
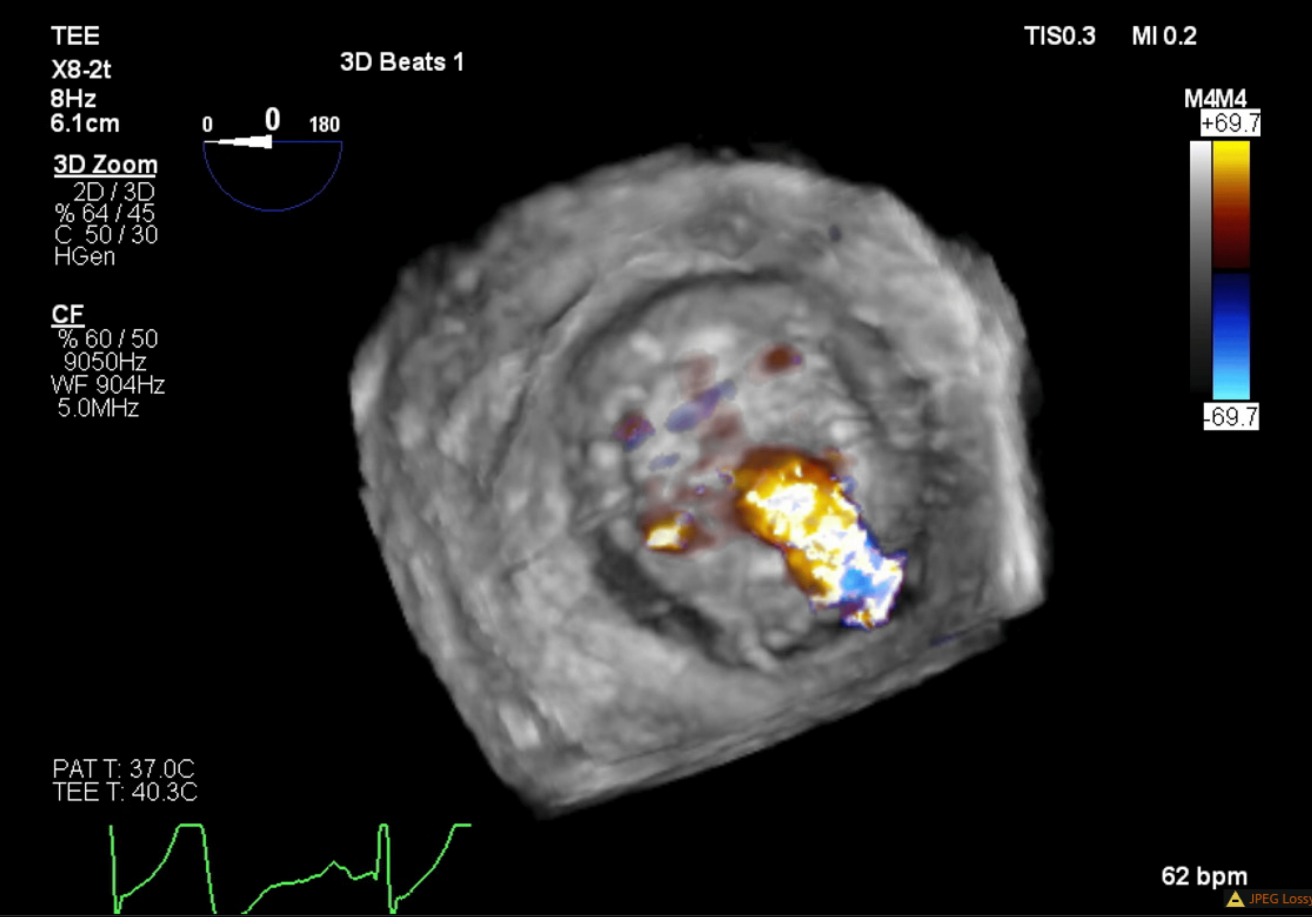
Transesophageal echocardiogram three-dimensional reconstruction with color Doppler, demonstrating paravalvular regurgitation originating from the posterior aspect of the mitral valve prosthesis.

Transesophageal echocardiography (TEE) confirmed severe PVL on the posterior aspect of the mitral valve prosthesis sewing ring (Figures 2-4). The valve appeared to be stable without rocking or dehiscence. She was diagnosed with mechanical hemolytic anemia secondary to severe PVL. 

Cardiac surgery and structural heart disease consultations were obtained. The patient was offered both redo-MVR and percutaneous repair. She proceeded with percutaneous repair with a 12 mm Amplatzer™ vascular plug device (Abbott Laboratories, Abbott Park, Illinois, United States). Post-procedural echocardiography demonstrated resolution of PVL. Subsequently, mechanical hemolysis was resolved and there were no further pRBC transfusions required.

## Discussion

PVL is a common complication in 7-17% of patients with MVR [4,5]. This often presents with symptoms of congestive heart failure (CHF) or hemolytic anemia. The cause of PVL is not always certain and is usually multifactorial. Common factors include dehiscence of sutures at the sewing ring, annular calcification, or other factors at the time of implantation [6]. PVL is roughly twice as common with mechanical valves compared to bioprosthetic valves [6]. Patients with mild PVL may often be asymptomatic, with increasing severity of PVL being associated with more severe symptoms. If a patient with a history of MVR develops symptoms of CHF or anemia, then PVL must be investigated. TTE or TEE is often used to establish a diagnosis [7].

Indications for repair are severe regurgitation, moderate regurgitation with CHF, or clinically significant hemolytic anemia. PVL repair is contraindicated in cases of endocarditis/vegetations or intracardiac thrombi that are at risk of being dislodged perioperatively, regurgitation area greater than one-third of the annulus area, and a rocking prosthesis [8]. These situations should warrant redo-MVR. 

Historically, surgical repair was associated with high mortality, about 7% intraoperatively, 17% mortality after one year, and 38% after five years. PVL recurrence is noted to be approximately 15% at five years and is a result of friable tissue and annular damage [9,10]. Conservative management carries significant risk, as regurgitation volume tends to worsen over time, exacerbating anemia and heart failure symptoms. For these reasons, the presence of PVL is associated with high morbidity and mortality. However, over the last 20 years, percutaneous closure of PVL has become the gold standard. Percutaneous closure is associated with much fewer perioperative complications and in-hospital mortality [11]. Candidacy for percutaneous closure requires a multidisciplinary evaluation that includes a cardiac surgeon, structural cardiologist, and imaging cardiologist [12].

Given that the current patient’s imaging showed moderate to severe regurgitation, no endocarditis, thrombi, or rocking prosthesis, she was considered a candidate for repair. Due to improved outcomes with percutaneous PVL repair, the patient was able to have a complication-free procedure one month after diagnosis and has been doing well since.

## Conclusions

Dark-colored urine is often assumed to be hematuria. In actuality, there is a broad differential diagnosis for dark colored urine, and it can result from a variety of urologic or non-urologic causes. Attention must be paid to the potential for non-urologic diagnoses when standard urologic testing is unremarkable. Hemolysis due to mechanical stress from PVL is a rare but important condition to identify expeditiously and may mimic hematuria. Early consideration for cardiac imaging in patients with a history of prosthetic valve replacement is recommended, as well as collaboration between cardiac, urologic, and hematologic specialties. Historically, PVL repair was performed surgically with high morbidity and mortality rates; however, the advent of transcatheter options has allowed for improved outcomes.
